# Environmentally Controlled Oscillator with Triplex Guided Displacement of DNA Duplexes

**DOI:** 10.1021/acs.nanolett.3c02176

**Published:** 2023-08-10

**Authors:** Qiuyan Huang, Jiyeon Kim, Kun Wang, Simon Vecchioni, Yoel P. Ohayon, Nadrian C. Seeman, Nataša Jonoska, Ruojie Sha

**Affiliations:** † Department of Chemistry, New York University, New York, New York 10003, United States; ‡ Department of Physics, New York University, New York, New York 10003, United States; § Department of Mathematics and Statistics, University of South Florida, Tampa, Florida 33620, United States

**Keywords:** DNA triplex, Toehold-assisted strand displacement, pH alteration, DNA origami

## Abstract

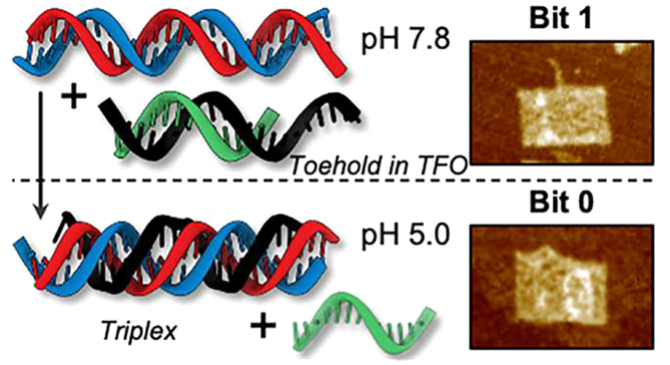

The use of DNA triplex association is advantageous for the reconfiguration of dynamic DNA nanostructures through pH alteration and can provide environmental control for both structural changes and molecular signaling. The combination of pH-induced triplex-forming oligonucleotide (TFOs) binding with toehold-mediated strand displacement has recently garnered significant attention in the field of structural DNA nanotechnology. While most previous studies use single-stranded DNA to displace or replace TFOs within the triplex, here we demonstrate that pH alteration allows a DNA duplex, with a toehold assistance, to displace TFOs from the components of another DNA duplex. We examined the dependence of this process on toehold length and show that the pH changes allow for cyclic oscillations between two molecular formations. We implemented the duplex/triplex design onto the surface of 2D DNA origami in the form outlining binary digits 0 or 1 and verified the oscillatory conformational changes between the two formations with atomic force microscopy.

DNA has become a highly versatile building block in the development of precise and predictable nanoscale structures with bottom-up assembly on the nanoscale. This is primarily due to its programmable characteristics and its ability to self-assemble through complementary base-pairing.^1−12^ The structural chemistry of DNA is most notably characterized by Watson–Crick base pairing.^13^ However, there exist other types of interactions among nucleic acids, one of the earliest observed being the triplex association between one polypurine and two polypyrimidine strands.^14^ The third pyrimidine nucleotide is attached to the purine in the major groove via Hoogsteen base pairing.^15^ The T-A-T base triplet can occur at neutral pH; however, in order to form a C-G-C base triplet, the pH must be lowered enough to protonate C in the major groove. A pH of 5.0–5.5 is typically sufficient for this protonation process. The requirement for a lower pH provides researchers the ability to control the in situ binding or unbinding of the triplex, and as a result, pH variation has been among the favored mechanisms to generate desired reconfigurations of DNA nanostructures, which have demonstrated potential applications as biosensors, molecular switches, logic gates, and for regulated drug delivery.^16−23^ Another widely used tool for conformational changes in DNA nanotechnology is toehold mediated strand displacement which has been used in reprogrammable nanodevices and complex reaction networks.^24−30^ Recently, researchers have successfully integrated the toehold-assisted strand exchange process into pH-dependent DNA triplex formation at both lower and neutral pH thereby attaining another layer of control.^10,31−34^ However, these toehold-mediated strand exchange reactions have only been explored by using DNA single strands as invasion strands to displace or replace TFOs on a given triplex substrate. In this study, we report that a DNA duplex with Hoogsteen complementarity to the toehold of the second duplex is capable of invading and displacing one strand from that duplex at lower pH by forming a DNA triplex.

Figure 1 illustrates the strand replacement process between the duplexes (D1–D2) and the duplex (TFO–D4). Upon lowering the solution pH to 5.0, the toehold segment of the TFO strand binds to the duplex (D1–D2) edge, forming a short triplex. Further strand invasion then occurs, resulting in the entire TFO strand attaching to the purine-rich strand D1 of the target duplex (D1–D2) and creating a triplex (D1–D2–TFO) while simultaneously displacing the short strand D4. Upon restoring the pH to 7.8, the TFO strand dissociates from the triplex and reattaches to the D4 strand, reforming the duplex (TFO–D4) and the duplex (D1–D2).

**Figure 1:**
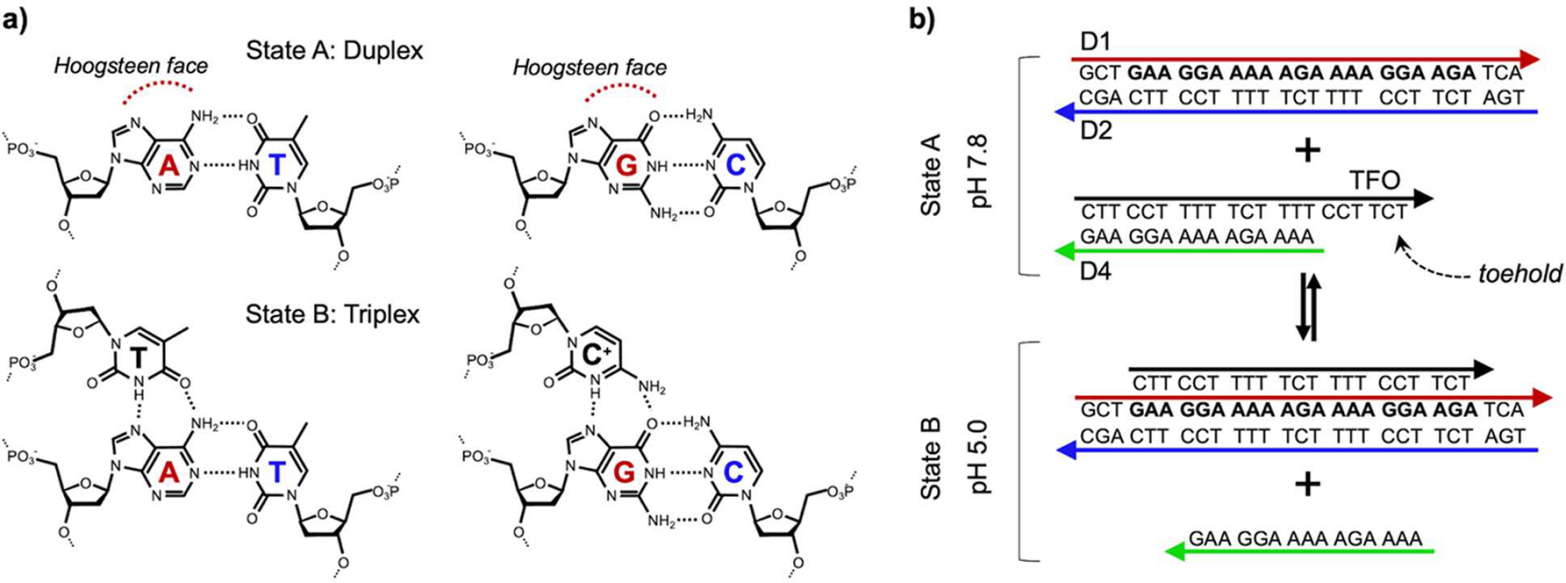
Concept and design: (a) Chemical structure of duplex and triplex DNA base pairs. (b) The scheme illustrates the strand displacements that occur under various pH conditions, with strand D1 highlighted in red, strand D2 in blue, the TFO strand in black, and strand D4 in green.

We performed nondenaturing polyacrylamide gel electrophoresis (PAGE) analysis for a duration of 16 h to evaluate the effect of different toehold lengths on the DNA triplex replacement process at pH 5.0. Specifically, we examined the duplexes (TFO–D4), (TFO–D5), and (TFO–D6), which contain toeholds of 6, 4, and 2 nucleotides (nt) in length, respectively. By comparing the results of the replacement process for all three duplexes, we sought to determine the optimal toehold length. As depicted in Figure 2, the duplex (TFO–D4) containing a 6 nt toehold exhibited the most efficient replacement with the duplex (D1–D2), evidenced by the absence of duplex bands in lane 2. However, the duplex (TFO–D5) with a 4-nucleotide toehold (lane 3) and the duplex (TFO–D6) with a 2-nucleotide toehold (lane 4) displayed partial or no replacement. It is clear that the degree of duplex–triplex replacement is dependent on the length of the toeholds.

**Figure 2:**
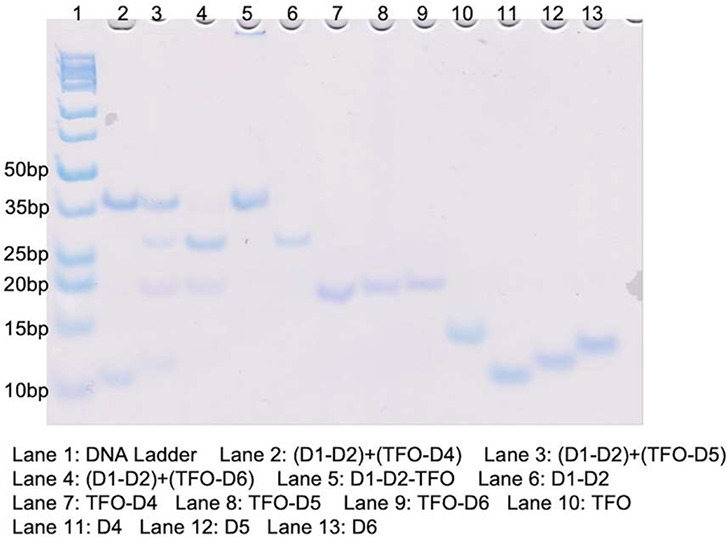
Toehold optimization by nondenaturing PAGE analysis of duplexes (TFO–D4), (TFO–D5), and (TFO–D6), with 6, 4, and 2 nt toeholds, respectively. Lane 2 shows the replacement between the duplex (D1–D2) and the duplex (TFO–D4); lane 3 shows the replacement between the duplex (D1–D2) and the duplex (TFO–D5); and lane 4 shows the replacement between the duplex (D1–D2) and the duplex (TFO–D6). Lane 5 shows the triplet (D1–D2–TFO) as the completed product. Component strands are shown in lanes 6–13. Replacement is carried out to completion for duplex (TFO–D4) (6 nt toehold, lane 2), partial completion for duplex (TFO–D5) (4 nt toehold, lane 3), and almost not at all for duplex (TFO–D6) (2 nt toehold, lane 4).

In Figure 3, we allowed approximately 2 h between each pH switch. The replacement process began in a pH 7.8 solution, and 10 μL of the resulting mixture was removed and added to Lane 2 of the pH 7.8 gel. The pH of the solution was then lowered to 5.0, and after a further 2-h waiting period, 10 μL was removed and added to Lane 2 of the pH 5.0 gel. The pH of the solution was then raised back to 7.8, and this process was repeated twice more until 10 μL was added to Lane 4 of the pH 5.0 gel. During the pH decrease to 5.0, strand D4 in the duplex (TFO–D4) was displaced, while the TFO strand displaced it to attach to the duplex (D1–D2), resulting in the formation of the triplex (D1–D2–-TFO) in which its yield was kept above 78% constantly in each cycle, shown in Table S2. When the pH of the solution was raised back to 7.8, the TFO strand reattached to the displaced D4 strand, reforming the duplex (TFO–D4) in which its yield was kept above 95% constantly in each cycle, shown in Table S2. These results demonstrate that the DNA triplex replacement process can be cycled at least three times and has the potential to go much further.

**Figure 3:**
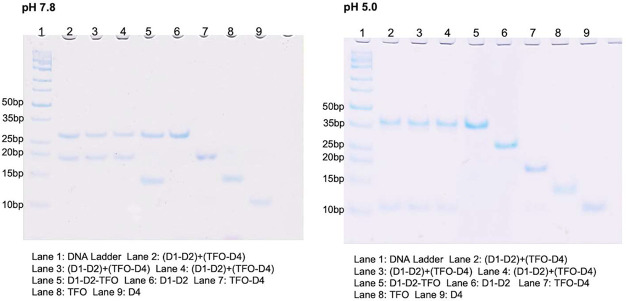
Nondenaturing PAGE analysis was conducted to assess the cycling ability of the DNA triplex replacement process. The left gel was run at pH 7.8, and the right gel at pH 5.0. Lanes 2–4 contain products from cycles 1–3 of the reaction. Lane 5 shows an unreacted triplex (D1–D2–TFO) and lane 6 shows the duplex (D1–D2). Lane 7 contains the duplex (TFO–D4), while lanes 8–9 contain the single strands TFO and D4, respectively.

Since the successful development of 2D DNA origami in 2006,^6^ the platform has been widely used as a substrate to characterize dynamic and responsive nanostructures using atomic force microscopy (AFM), a high-resolution imaging technique that provides valuable information on the properties of nanoscale materials.^35−38^ In this study, we utilized a similar approach to construct rectangular DNA origami with duplex–triplex interconversion devices on its surface as a one-bit memory element. As shown in Figure 4, at pH 7.8, the anchoring sites containing DNA duplexes (H1–H2–H3) are positioned on the left side of the DNA origami, while the other DNA duplex (H4–H5) is present in the solution. Digit 1 can be observed using AFM due to the height difference generated by the duplex (H1–H2–H3) bridging the two anchoring sites. At pH 5.0, the duplex (H4–H5) invades the duplex (H1–H2–H3) by utilizing the single-stranded portion of the duplex (H1–H2–H3) as the toehold, displacing strand H3, and forming a triplex (H4–H5–H3). This process leaves only two single strands, H1 and H2, on the anchoring sites, which are not visible with AFM due to their flexibility and insufficient height difference. To showcase the change of the local environment on the DNA origami surface, we placed a conventional DNA triplex on the right side of the origami as the control. At pH 5.0, triplex (H6–H7–H8-H9) bridges two anchoring sites and is visible with AFM. Upon switching to pH 7.8, triplex (H6–H7–H8-H9) dissociates, duplex (H8–H9) is released from the DNA origami, and only two single strands, H6 and H7, remain attached to the anchoring sites, which are not visible with AFM.

**Figure 4:**
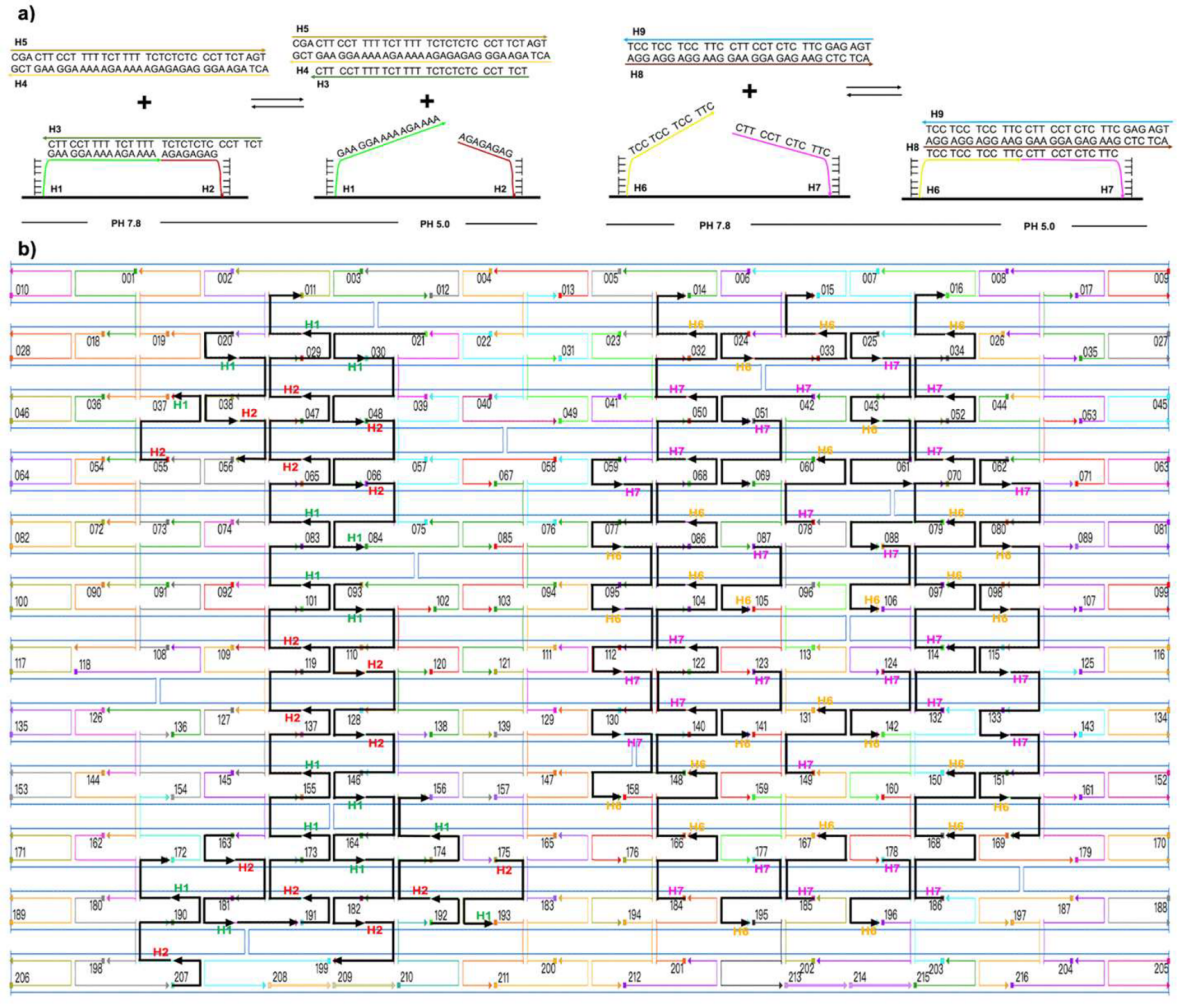
Design of duplex–triplex interconversion memory devices on 2D DNA origami. (a) Left: On the surface of the origami, strands H1 and H2 are utilized as anchoring sites. At pH 7.8, the strands H1, H2, and H3 combine to form a duplex (H1–H2–H3), which bridges the two anchoring sites and is detectable with AFM. When the pH is lowered to 5.0, strand H3 is displaced by a duplex (H4–H5) to form a triplex (H4–H5–H3), causing the sites to become undetectable with AFM. Right: strands H6 and H7 are utilized as anchoring sites. At pH 5.0, H6, strands H7, H8, and H9 form a triplex (H6–H7–H8–H9), which bridges the two anchoring sites and can be observed with AFM. When the pH is switched back to 7.8, the duplex (H8–H9) is released from DNA origami, rendering the sites undetectable with AFM. (b) Pattern design on origami: On a rectangular DNA origami measuring 100 × 70 nm, we have constructed the 0 and 1 digits using specific anchor points. More specifically, H1 (green) and H2 (red) were utilized as the anchoring sites for Digit 1, with a total of 16 pairs of them. On the other hand, for Digit 0, H6 (yellow) and H7 (purple) were used as the anchoring sites, consisting of 28 pairs of them.

Our study employed AFM to read out the state of duplex–triplex interconversion devices on DNA origami at both pH 7.8 and pH 5.0. Our findings indicate that at pH 7.8, over 90% of DNA origami displayed the expected digit 1 pattern, with no digit 0 present, as shown in Figure 5a. However, upon switching to pH 5.0, approximately 40% of the DNA origami experienced instability and folded in on themselves, rendering pattern detection on their surface impossible. Nevertheless, of the remaining DNA origami with clear patterns, over 90% displayed the expected digit 0 pattern and no digit 1 at pH 5.0, as shown in Figure 5b. To further validate these results, we examined DNA origami with only single strands H1, H2, H6, and H7 on the anchoring sites by AFM, and no discernible pattern was detected, as shown in Figure S1. This provides evidence that the patterns showing digits 1 and 0 are generated by the formation of either duplex or triplex structures that bridge the two anchoring sites. Additionally, we demonstrated the reversibility of duplex–triplex interconversion by switching the pH back and forth between 7.8 and 5.0, as shown in the AFM images of four stages in Figure S2 and the effective yields of DNA origamis with the expected pattern in each cycle are above 90%, shown in Table S3. Our study provides evidence that this process can be cycled on DNA origami, much like it can in solution, in order to form stable memory elements with composable parts.

**Figure 5:**
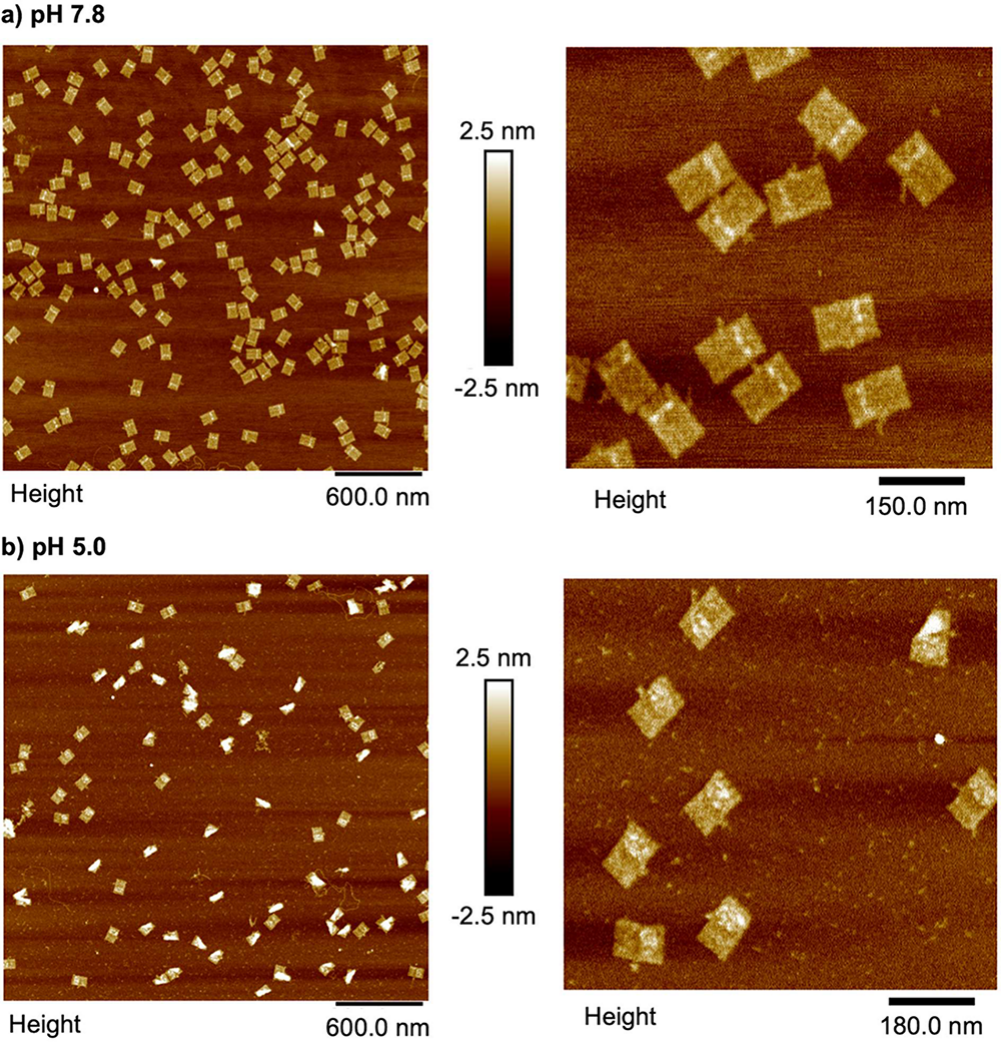
AFM image of DNA origami. (a) The left panel of the image displays a 5 μm × 5 μm field of DNA origami, with a close-up zoom inset. At a pH of 7.8, digit 1 is discernible, whereas digit 0 is not. (b) The left panel of the image depicts a 5 μm × 5 μm field of DNA origami, while the right panel provides a zoomed-in view. At pH 5.0, digit 0 is discernible on the origami’s surface, whereas digit 1 is not visible.

In this study, we have successfully demonstrated the occurrence of duplex–triplex interconversion both in solution and on the surface of DNA origami by means of pH alteration. A key finding is that a single-stranded toehold can facilitate the invasion of one structurally rigid DNA duplex by another, leading to the displacement of the TFO strand and the formation of a rigid DNA triplex at lower pH. Notably, this represents the first evidence that triplex base pairing can displace duplex base pairing without compromising the rigidity and integrity of the DNA structures. The use of toehold sequences in combination with pH variation offers an additional level of control and addressability, which bodes well for the design of more complex systems of DNA reconfiguration and nanodevices.

Finally, we leveraged this reconfigurability to engineer a 1-bit memory element with a simple scanning probe readout. This device demonstrates a proof-of-concept for more complex circuits that store the outputs of chemical reactions through pH-encoded duplex and triplex systems. A key element of our duplex/triplex reaction network lies in the ability of two itinerant duplexes to mutually exchange components among single-stranded, duplex, and triplex states in an isothermal system. Our system shows that control can be achieved at both the sequence level and also through changes in the environment (here pH). The oscillatory property of the system also shows environmental (rather than strand-initiated) control of the molecular dynamics. Because the pH-based initiation of the duplex/triplex transition happens simultaneously at all configurations, this control suggests possible stages in the molecular configurations and controlled dynamics of structures. Optimization of origami substrates, pattern density, and reaction scaling may ultimately lead to complex surface- and solution-based environmentally controlled memory systems for nanotechnological applications.

## Supporting Information Available

Materials and methods; DNA sequences; Yields of targeted products of each cycle in solution; Effective yields of DNA origamis with expected pattern in each cycle; AFM image of control DNA origamis; AFM images of cycling of duplex–triplex interconversion devices on DNA origami (PDF)

## Dedication

^∥^ Dedicated to the memory of Prof. Nadrian C. Seeman, who passed away during the preparation of this manuscript on November 16, 2021.
