# Supplementary material for: Environmentally Controlled Oscillator with Triplex Guided Displacement of DNA Duplexes
Source: Nano Lett. 2023 Aug 10;23(16):7593–8. doi: 10.1021/acs.nanolett.3c02176 (PMC10450806; doi:10.1021/acs.nanolett.3c02176)
Supplement: Supplementary file 1 — nl3c02176_si_001.pdf [file nl3c02176_si_001.pdf]

Supporting Information: Environmentally Controlled Oscillator with Triplex Guided Displacement of DNA Duplexes

Qiuyan Huang[1#], Jiyeon Kim[1#], Kun Wang[2#], Simon Vecchioni[1], Yoel P. Ohayon[1], Nadrian C. Seeman[1†], Nataša Jonoska[3*] and Ruojie Sha[1*]

[1]Department of Chemistry, New York University, New York, NY 10003, USA.
[2]Department of Physics, New York University, New York, NY 10003, USA.
[3]Department of Mathematics and Statistics, University of South Florida, Tampa, FL 33620, USA
[#]These authors contributed equally
†Deceased
*Corresponding Author; email: jonoska@mail.usf.edu and ruojie.sha@nyu.edu

Materials and Methods

Purification of DNA Strands

All the DNA strands were purchased from Integrated DNA Technologies. DNA strands were dissolved in denaturing tracking dye buffer (90% formamide with 10 mM sodium hydroxide, 1 mM EDTA, xylene cyanol FF, bromophenol blue) and purified through 20% denaturing PAGE (40% acrylamide [Acrylamide/Bisacrylamide 19:1], 50% urea, TBE buffer, which contains 89 mM Tris, 89 mM Boric Acid, and 2 mM EDTA). The appropriate bands were cut from the gels, eluted in buffer (200 mM ammonium acetate, 10 mM magnesium acetate, 2 mM EDTA), extracted by butanol, and precipitated by ethanol.

Design of the Duplexes

We tested 3 duplexes with different toehold lengths (TFO-D4, TFO-D5, and TFO-D6 as shown in Table S1) through non-denaturing PAGE to determine which would act as the best replacement by using toehold-mediated strand displacement. When strand D4, D5 or D6 bound to the TFO strand, duplexes with 6-, 4-, and 2-nucleotide long toeholds formed respectively.

Annealing of Duplexes

The different duplexes, D1-D2, TFO-D4, TFO-D5, and TFO-D6, were formed in separate tubes (each at a 10 μM concentration). For every tube, a TAE/Mg buffer containing 40 mM Tris, 2 mM EDTA, and 12.5 mM magnesium acetate was used to dilute the mixture, and each duplex was annealed over 1 hour starting from 90˚C to 65˚C to 45˚C and ending at 37˚C for 5, 15, 20 and 20 minutes respectively.

Formation of Control Triplex Complexes

The TFO strand with a concentration of 100 μM was used in the formation of the control triplex complex containing a 6-nucleotide long toehold. The strand was diluted with TAE/Mg buffer containing 40 mM Tris, 2 mM EDTA, and 12.5 mM magnesium acetate. 1 μL of the TFO solution was added to the D1-D2 duplex at 4°C and left overnight. Under acidic pHs, triplex formation is favored.

Preparation of DNA Duplexes and Native PAGE Analysis

Native PAGE was used to confirm the replacement reaction. We prepared two 15% native PAGE at 2 different pHs: 7.8 and 5.0. A GeneRuler Ultra Low Range DNA Ladder was used as a marker. The gel was

run at 10 V/cm at room temperature. Both gels were visualized using Stains-All dye. The D1-D2, TFO-D4, TFO-D5, and TFO-D6 duplexes were prepared with both pH 5.0 and 7.8 1xTAE buffers and used to find the optimal toehold length for the replacement. After we concluded that the TFO-D4 duplex made the most effective replacement (Figure 2 at Lane 2), we made 50 µL of both the D1-D2 duplex and the TFO-D4 duplex using a pH 7.8 1xTAE buffer. These were then combined, and a 10 µL aliquot of this mixture was taken for native PAGE analysis. Separate solutions of 1.0 M hydrochloric acid and 1.0 M sodium hydroxide were used either to lower or to raise the pH. We allowed time for migration to occur after every pH switch.

Preparation of DNA rectangular origami

M13mp18 from Bayou Biolabs (10 nM) and staple strands from IDTDNA (100 nM) are mixed in 100 uL 1xTAE/Mg buffer and incubated from 70^0C to 20^0C at a ramp of 7^0C/hour to form DNA rectangular origami. After the excess staple strands were filtered out by using Amicon Ultra Centrifugal filter (100K), the concentration of DNA origami was measured with Implen Nanophotometer.

AFM imaging of DNA origami

5 µL of DNA origami sample was deposed onto fresh mica surface (Ted Pella, Inc.) and was left for 1 min to be absorbed. Rinse the mica with 30 µL double-distilled water for three times and blow dry by using compressed air. All AFM imaging was performed on a MultiMode 8-HR atomic force microscope and NanoScope 6 Controller (Digital Instruments) from Bruker in 'ScanAsyst in air' mode. Model of AFM Probes is SCANASYST-AIR.

Pattern generation on the DNA origami surface

The strands involving in producing pattern '1' or '0' were mixed with the filtered DNA origami and kept at room temperature for 2 hours. The final concentration of origami was 2nM. The strands involving in producing pattern '1' or '0'should be 10 time to its bind sites, 16 binding sites for pattern 1 and 28 binding sites for pattern 0, so the final concentration of strands involving in producing pattern '1'was 320nM and for pattern '0'was 560nM. Separate solutions of 1.0 M hydrochloric acid and 1.0 M sodium hydroxide were used to lower or to raise the pH and allowed 2h for interconversion to occur after every pH switch. The pattern '1'would show up on origami while the pH of TAE buffer was 7.8, and pattern '0' would show up while pH is 5.0.

Sequences for PAGE experiment.

Name	Sequences	Bases
D1	GCT GAA GGA AAA AGA AAA GGA AGA TCA	27
D2	TGA TCT TCC TTT TCT TTT TCC TTC AGC	27
TFO	CTT CCT TTT TCT TTT CCT TCT	21
D4	AAA AGA AAA AGG AAG	15
D5	GGA AAA GAA AAA GGA AG	17
D6	AAG GAA AAG AAA AAG GAA G	19

Staple strands for DNA origami

Name	Sequences	Bases
SS001	TTC TTT GAT TAG TAA TTA TCG GCC TTG CTG GTA CAC GAC C	40
SS002	GCA AAT TAT TAC CGC CAG CCA TTG ATG GAT TA	32
SS003	GAG GCC ACC ATG GAA ATA CCT TTC CAG TCG GG	32
SS004	CCA GAA TCC GTG CCA GCT GCA TTA AGC TAA CT	32

SS005	GAA AAA CCG CGG GGA GAG GCG GTT TAA AGT GT	32
SS006	ATT AAA GAG GGT GGT TTT TCT TTT CAC AAT TC	32
SS007	AGT GTT GTA ACA GCT GAT TGC CCT TAG CTG TT	32
SS008	ATA AAT CAA GAG AGT TGC AGC AAG GGG TAC CG	32
SS009	TTT TAT CCT GTT TGA TGG TGG CCC CAG CAG GCG AAA TTT T	40
SS010	TTT TGT AGA AGA ACT CAA ACA ACA TCA CTT GCC TGA TTT T	40
SS011	AAA ACG CTC GAG TAA AAG AGT CTG TCC ATC AC	32
SS012	AAA CCT GTC TGA GAA GTG TTT TTA TAA TCA GT	32
SS013	GCC AAC GCG TCT ATC AAG ACA GGA ACG GTA CG	32
SS014	GGG CGC CAA CGT GGA CTC CAA CGT CAA AGG GC	32
SS015	AGA CGG GCT CCA GTT TGG AAC AAG AGT CCA CT	32
SS016	TGG CCC TGA AAG AAT AGC CCG AGA TAG GGT TG	32
SS017	GCT GGT TTG TTC CGA AAT CGG CAA AAT CCC TT	32
SS018	AGT AAT AAT TCT GAC CTG AAA GCG AAC TAA TA	32
SS019	CAC CAG TCA ATA TCC AGA ACA ATA ACC GTT GTA GCA ATA C	40
semi20	TTTACATTAGACAATA	16
SS022	CAC ATT AAT TAA AAA TAC CGA ACG AAA TAT CA	32
SS023	AAA GCC TGT AAA ACA GAG GTG AGG GAA AAA TC	32
semi25	TCC TGT GTG TGC TGC A	16
SS026	AGC TCG AAG GGT TTT CCC AGT CAC AGC GCC AT	32
SS027	TTT TGC ATG CCT GCA GGT CGG GCC AGT GCC AAG CTT TTT T	40
SS028	TTT TAA CAG AGA TAG AAC CCA AGG GAC ATT CTG GCC TTT T	40
29	CGTCTGAACAACAGGA	16
SS030	ACT GAT AGT TGG CAA ATC AAC AGT TTA AAA GT	32
SS031	GCA GAA GAG GGT GCC TAA TGA GTG ATG AAT CG	32
semi32	CG GAA GCA TGC GTA TT	16
semi33	TA TCC GCT CAC CAG TG	16
semi34	CA TGG TCA TCA CCG CC	16
SS035	AAA ACG ACA CTC TAG AGG ATC CCC CGG TCC AC	32
SS036	GAT TAG AGA GTA TTA GAC TTT ACA AAT AAT GG	32
SS037	GCA CTA ACT AAG AAT ACG TGG CAC GGC AGA TT	32
semi38	TCCTTTGCATATAATC	16
SS039	CTG GTC AGC CCT AAA ACA TCG CCA TTG CGT TG	32
SS040	AAC CCT CAA ACA AAG AAA CGA GCG AGT AAC AA	32
SS041	TAA AGC ATA TTC TCC GTG GGA ACA GGC CTT CC	32
43-61	AC CGT GCA AAA TTT TTG TTA AAT CGA AAA CAA AAT TGA AC	32
SS044	TCG CCA TTG ACG ACG ACA GTA TCG GTA AAC GT	32
SS045	TTT TCG GCA CCG CTT CTG GTA CTC CAG CCA GCT TTC TTT T	40
SS046	TTT TCA TTT GAG GAT TTA GAC CGT CAA TAG ATA ATA TTT T	40
SS049	CCC GTC GGC ACC TTG CTG AAC CTC AAC CAC CA	32
SS053	AAG ATC GCG CCG GAA ACC AGG CAA GAC GTT GT	32
SS054	AAG GGT TAA ACA GAA ATA AAG AAA AAT CAT AG	32
SS056	CTG ATT GTG TTT AAC GTC AGA TGA ACG CTG AG	32
SS057	ATC ATC ATC ATT ATC ATT TTG CGG ATC AAT AT	32
SS058	CAA CAT TAA ATG TCA CCA GAA GGA GCC TGA TT	32
semi59	TGT AGC CAC GCG CAG A	16
SS060	TTT TAA CCG TTG GTG TAG ATG GGC TGC GGG CC	32
79-61-70	ACA AGA GA G GTA ATC G TAA AAC TAT GTT AAA A	32
semi62	TAA TAT TTG CAT GTC A	16
SS063	TTT TAC AGG AAG ATT GTA TAC AGA AAA GCC CCA AAA TTT T	40
SS064	TTT TAA TTA TTT GCA CGT AAG AAC CTA CCA TAT CAA TTT T	40
83-65	AGATTAAGATATACAG TAACAGTATGATGGCA	32
SS067	GCT TTG AAA ATC GTC GCT ATT AAT AGC CTT TA	32
86-68	CA TAA ATC ATT CAT TT CAA TTA CCA AAA TAA T	32
SS069	AGA AGA TGT TAC CTT TTT TAA TGG GAG TAA TG	32
SS071	TTG ATA ATA GCA AAT ATT TAA ATT GCC TCA GG	32
SS072	GTC TGA GAT TAT ATA ACT ATA TGT ATA AAC AC	32
SS073	TTT ATC AAT TGC GTA GAT TTT CAG TTG GAT TA	32
SS074	AAG AGT CAC CAA TCG CAA GAC AAA CGA CCG TG	32

SS075	CCT TAG AAA AAC AAT AAC GGA TTC GCG GAA TT	32
SS076	CTT CTG TAT ACC AAG TTA CAA AAT GCT TTC AT	32
semi79	A TCG ATA ATT ACA TTT AGA AAG GC	24
SS081	TTT TCT ATT TTT GAG AGA TCA TGC CGG AGA GGG TAG TTT T	40
SS082	TTT TTC CGG CTT AGG TTG GGG ACT ACC TTT TTA ACC TTT T	40
SS084	ATA TTT TAG TTA ATT TGC GGG AGA TAA TTT TC	32
SS085	TTT CAA CGC CAA AAA CAT TAT GAC CAG AGG CA	32
SS089	ATA AAT TAT ACA AAG GCT ATC AGG TAC CCC GG	32
SS090	CGG AAT CAT GCG TTA TAC AAA TTC TAT TTT CAT CGT AGG A	40
SS091	AAA TAA GAA AAT GCT GAT GCA AAT ATA GTG AA	32
SS092	TGA TAA ATA ACG CTC AAC AGT AGG ACC GCA CT	32
93-110	AACAACGCTTATCATT CCAAGAACTCAAAAAT	32
SS094	CGG TTG TAC AAG GAT AAA AAT TTT TAA CCT TG	32
95-112	CC GAC AAA AAA AAT AA TAT CCC ATT AAG AGG A	32
SS096	TCA TAC AGA AGA TTC AAA AGG GTG AAC AAT TT	32
98-115	AT TTA GTT TCA ACA TG TTT TAA ATG TAC CTT T	32
SS099	TTT TAA CCT GTT TAG CTA TAT TCG CAA ATG GTC AAT TTT T	40
SS100	TTT TCC TGT TTA GTA TCA TAT AAT TAC TAG AAA AAG TTT T	40
SS102	AAT TTA GGC CTG TAA TAC TTT TCA TCT TCT GA	32
SS103	TTT TCG AGG TAG AAA CCA ATC AAT GTC AGA AG	32
SS105	ACG ACA ATT GTT TAT CAA CAA TAG GTT TTA AT	32
SS106	AAC AGT TGG TGT CTG GAA GTT TCA GGA AGC AA	32
SS107	AGA TAC ATT TTT CAT TTG GGG CGC TCT AGC TG	32
SS108	GCC GTT TTT TAC CAG TAT AAA GCC AAG GCG TT	32
SS109	CAT CGA GAT AAA CAG TTC AGA AAA ATC GTC AT	32
SS111	ACG AGC ATC CAG TAA TAA GAG AAT AGC TAA AT	32
SS113	AAC GCG CCA AAC AAC ATG TTC AGC CCA ATA AA	32
SS116	TTT TTA GAG CTT AAT TGC TGA TTT TTG CGG ATG GCT TTT T	40
SS117	TTT TAT AGC AAG CAA ATC AGA TCA TTA CCG CGC CCA TTT T	40
SS118	ATA TAG AAG GCT TAT CCG GTA CTC AAA TGC TTA CAA GCA A	40
SS120	CTA TTA TAA ATC GGC TGT CTT TCC CAA CAT GT	32
SS121	CAA AGC GGA AGA AGT TTT GCC AGA CCA GTT AC	32
SS125	AAG AGG TCA ATA TAA TGC TGT AGC TGA CCA TT	32
SS126	AGG CGT TTA GCC TTA AAT CAA GAT GGT AAT TG	32
SS127	AAA TAT TCC CCA GCT ACA ATT TTA GAA TTA AC	32
SS129	TTT TGC AAA TTG CAT CAA AAA GAT CCT AAT TT	32
148-130-140	GTA AAT TG GGT TTA AT TTC AAC TTC GTT TAC C	32
SS131	TAA GAG CAC AAA GCG AAC CAG ACC TAA TGC AG	32
SS134	TTT TCA GGT AGA AAG ATT CAC GGA ACA ACA TTA TTA TTT T	40
SS135	TTT TTT GCG GGA GGT TTT GAT AGC GAA CCT CCC GAC TTT T	40
SS136	ATT TTG CAA TTG AAT CCC CTT CTA AGA ACG CG	32
155-137	AGACGGGATCCTGAAT CTTACCAACGTCCAAT	32
SS138	CTA ATT TGG GGG GTA ATA GTA AAA TAC CCT GA	32
SS139	AAA ATA AAA AAA TGA AAA TAG CAG CGC GAA AC	32
SS141	GTG AAT TAT GAC GAG AAA CAC CAG TGC TCC AT	32
SS142	TCA TTA TAA AAG CTG CTC ATT CAG GAC GGT CA	32
SS143	CGA ACT AAT CAG TTG AGA TTT AGG CTT TTG AT	32
SS144	AGC GCT AAA GCC CAA TAA TAA GAG AAC GCA AT	32
SS145	TGA ACA CCA GCA ATA GCT ATC TTA GCC GAA CA	32
SS147	TAA CGT CAC AGC CAT ATT ATT TAT CGA GAG GC	32
SS150	AAC GTA ACC CAG TCA GGA CGT TGG AAT GCA GA	32
semi151	AG AGG ACA GGG ATC GT	16
SS152	TTT TGG CTG ACC TTC ATC AAA CCA GGC GCA TAG GCT TTT T	40
SS153	TTT TCA CAA GAA TTG AGT TAT ATC AGA GAG ATA ACC TTT T	40
SS154	AAT GAA ATC TGA ACA AAG TCA GAG TAG TTG CT	32
SS156	ATA CCA AGC CTT TAC AGA GAG AAT TCC AGA GC	32
SS157	AAA GTA CAA CGA AGG CAC CAA CCT GTC ACA AT	32
SS158	TAA ATT GTT CCA TTA AAC GGG TAA CAG CGC CA	32
SS159	GTT ACT TAT TGA GGA CTA AAG ACT GAT TGA GG	32

SS160	ATC ATA AGT CGG AAC GAG GGT AGC ATT ATT CA	32
SS161	GTG TAC AGG AGT AAT CTT GAC AAG TAA TAA AA	32
SS162	AAT AAC GGC TTA TTA CGC AGT ATG GAG CCA CC	32
semi163	AAGTTACCATACATAC	16
164-182	GACACCACATTTTCGG TCATAGCCCGTCTTTC	32
SS165	ATG CCA CTA CGG AGA TTT GTA TCA TTT TTG TT	32
SS167	CAG AGG CTG CCG GAA CGA GGC GCA TGA ATA AG	32
SS168	AGA CAG CAG GAA CCG AAC TGA CCA CCC AAA TC	32
SS169	CAC CCT CAC GAC TTG AGC CAA CCA TCG CCC AC	32
SS170	TTT TGA GGC TTG CAG GGA GTG ATA TAT TCG GTC GCT TTT T	40
SS171	TTT TGG CAT GAT TAA GAC TCA ATA CCC AAA AGA ACT TTT T	40
SS172	CGT AGA AAA GAA GGA AAC CGA GGA CAA GAA AC	32
semi174	AGAGGCAACAGCGATT	16
SS176	AAG ACA AAC GTA ATC AGT AGC GAC TTC AGC GG	32
SS179	GCA TAA CCT AAA GGC CGC TTT TGC GAT GAA CG	32
SS180	ACC GGA ACC CAC CCT CAG AGC CAC GAG GTT GA	32
semi181	CACCGTAACGATCTAA	16
SS183	GTT TGC CTA ATT CAT ATG GTT TAC AAT ACG TA	32
SS187	CGA CAA TGA CAA CAT TTG GGA ATT CTT TAA TT	32
SS188	TTT TAC AGC TTG ATA CCG ATG AGG TGA ATT TCT TAA TTT T	40
SS189	TTT TGC CAC CCT CAG AAC CGC GCC TCC CTC AGA GCC TTT T	40
semi190	CGGAACCATTAGCAAA	16
semi191	AGTTTTGTCCCTTATT	16
semi192	AATTTTCTGCGCGTTT	16
SS193	TTT TGC TAA ACA CTG AGT TTC GTC AAT AAG TT	32
SS194	AGT GAG AAG ATA GCA AGC CCA ATA ACA GTG CC	32
SS195	GCG AAT AAC CAC CCT CAG AGC CAC CCT ATT TCG GAA CCT A	40
SS196	CAA AAA AAC GCC ACC CTC AGA ACC GCC ACC CT	32
SS197	GTA TCG GTA GGT GTA TCA CCG TAC GGA TTA GG	32
SS198	GGC AGG TCA ATC CTC ATT AAA GCC AGA ATG GA	32
SS199	ACA GAC AGC CCT CAT AGT TAG CAC CAG AGC CGT CTC TGA ATT TAC CGT	48
SS200	TGT ACC GTA ACA ACT TTC AAC AGT AGA ATC AA	32
SS201	TTT TCA GGT AGA AAG GAA CAA CTA CCA TCG AT	32
SS202	CAG AAC CGT AAT TTT TTC ACG TTG CAT TAG CA	32
SS203	TTT AGT ACA GGC TCC AAA AGG AGC AGA GCC AG	32
SS204	TTT TCC CGG AAT TTA TCA GCT TGC TTT CAG TTG CGC TTT T	40
SS205	TTT TTC GAG AGG GTT GAT ATA GGC GGA TAA GTG CCG TTT T	40
SS206	TAT TCA CAA ACA AAT AAG ACG ATT GGC CTT GA	32
semi207	AAGCGCAGCCGCCAGC	16
SS208	TCC AGT AAG CGT CAT A	16
SS209	CAT GGC TTT TGA TGA T	16
SS210	ACA GGA GTG TAC TGG TAC CAG TAC AAA CTA CAA GTA AAT G	40
SS211	TTA ACG GGG TCA GTG CCT TGA GTA GGA ACC CA	32
SS212	CGT ATA AAC AGT TAA TGC CCC CTG CAC CCT CA	32
SS213	TTA TTC TGA AAC ATG A	16
SS214	AAG TAT TAA GAG GCT G	16
SS215	AGA CTC CTC AAG AGA ATC AGG AGG	24
SS216	ATT AGC GGG GTT TTG CTC AGT ACC AAG TAT AG	32

Sequences involved in producing pattern ‘1’ and ‘0’.

Name	Sequences	Bases
H2-47-29-H1	AGA GAG AG TTT TTT ATTGAGGATGGCTATT AGTCTTTACGCTCAAT TTT TTT GAA GGA AAA AGA AAA	67
H2-65-47	AGA GAG AG TTT TTT ATTCATCACCGAACGT TATTAATTTGAAAGGA	46
101-83-H1	TGAAATACGAACGCGA GAAAACTTGATAGCTT TTT TTT GAA GGA AAA AGA AAA	53

H2-119-101-H1	AGA GAG AG TTT TTT AACCAAGTGCTTAATT GAGAATCGTAATGGTT TTT TTT GAA GGA AAA AGA AAA	67
H2-137-119	AGA GAG AG TTT TTT ACTGCGGACGAGAATG ACCATAAAGGGTATTA	46
173-155-H1	AGCAGATACCGAAGCC CTTTTTAAAGCGCATT TTT TTT GAA GGA AAA AGA AAA	53
H2-191-173-H1	AGA GAG AG TTT TTT AGCGTTTGGGCAACAT ATAAAAGAGAAAAGTA TTT TTT GAA GGA AAA AGA AAA	67
21-H1	CGCTCACTGCCCGCTA CATTTTGAATGCGCGA TTT TTT GAA GGA AAA AGA AAA	53
H2-48	AGA GAG AG TTT TTT TTGAGTAAATTCCTGA TTATCAGACCTTTTAC	46
H2-66-H1	AGA GAG AG TTT TTT ATCGGGAGTCCTTGAA AACATAGCTTTCAAAT TTT TTT GAA GGA AAA AGA AAA	67
93-H1	CCTAAATTCCATATTT TTT TTT GAA GGA AAA AGA AAA	37
H2-110-128	AGA GAG AG TTT TTT CAGGTCTTTGTTTAGA CTGGATAGCGCTAACG	46
H2-128-146-H1	AGA GAG AG TTT TTT AGCGTCTTAACATAAA AACAGGGAACTCATCT TTT TTT GAA GGA AAA AGA AAA	67
146-164-H1	TTGACCCCAAGAATAC ACTAAAACAACGCAAA TTT TTT GAA GGA AAA AGA AAA	53
H2-182	AGA GAG AG TTT TTT CAGACGTTACGCCTGTAGCATTCC	46
H2-55-H1	AGA GAG AG TTT TTT TACTTCTGAACAATTCGACAACTCCTTTAGGA TTT TTT GAA GGA AAA AGA AAA	67
H2-207-190-H1	AGA GAG AG TTT TTT ATTGACAGCACCCTCA GAGCCGCCAAAATCAC TTT TTT GAA GGA AAA AGA AAA	67
H2-20-38-H1	AGA GAG AG TTT TTT TTTTTGAAAGGTTATC TAAAATATGTATTAAA TTT TTT GAA GGA AAA AGA AAA	67
H2-163-181-H1	AGA GAG AG TTT TTT ATAAAGGTCCATCTTT TCATAATCACCAGAAC TTT TTT GAA GGA AAA AGA AAA	67
H2-192-174-H1	AGA GAG AG TTT TTT TCATCGGCGGAATAAG TTTATTTTAAAACGAA TTT TTT GAA GGA AAA AGA AAA	67
H2-175-H1	AGA GAG AG TTT TTT CAATAGAATTAGCGTC AGACTGTAGTATGGGA TTT TTT GAA GGA AAA AGA AAA	67
H7-59-77-H6	CTT CCT CTC TTC TTT TTT GG CGA ATT AAT ATA TG TGA GTG AAT AGA ACC C TTT TTT TCC TCC TCC TTC	68
77-95-H6	TC ATA TAT AAG CCT CA GAG CAT AAA TAA AGT A TTT TTT TCC TCC TCC TTC	50
H7-112-130	CTT CCT CTC TTC TTT TTT AG CCC GAA ATA AAA AC CAA AAT AGC CCA ATC C	50
H7-130-148-H6	CTT CCT CTC TTC TTT TTT AA AGA GAT G GCT TTA AGA AAC GAT TCG CCT GA TTT TTT TCC TCC TCC TTC	68
H7-50-32-H6	CTT CCT CTC TTC TTT TTT CA GCA AAT CGG TCA GT ATT AAC ACA TAC GAG C TTT TTT TCC TCC TCC TTC	61
H7-68-50	CTT CCT CTC TTC TTT TTT TC GCG TCT AAC GGC GG ATT GAC CGA GAG CCA G	46
104-86-H6	AA GCA ATA TTT AAA TG CAA TGC CTA AAC AGT A TTT TTT TCC TCC TCC TTC	50
H7-122-104-H6	CTT CCT CTC TTC TTT TTT TG AAC AAG AGG TAA AG TAA TTC TGG CAA AAT T TTT TTT TCC TCC TCC TTC	68
H7-140-122	CTT CCT CTC TTC TTT TTT AG ACG ACG AGA CTT CA AAT ATC GCA TAA GTC C	50
166-H6	AGG AAG TTG TCG AAA TCC GCG ACC AAC GAG TA TTT TTT TCC TCC TCC TTC	50
H7-184-H6	CTT CCT CTC TTC TTT TTT AGC AGC ACA GGG CGA CAT TCA ACC TTT TCA TG TTT TTT TCC TCC TCC TTC	68
24-H6	CAC ACA ACC GCC TGC A TTT TTT TCC TCC TCC TTC	34
H7-51	CTT CCT CTC TTC TTT TTT TAG GTC ACA ATA GGA ACG CCA TCA TGA GCA AA	50
H7-177-H6	CTT CCT CTC TTC TTT TTT GAG GGA AGA ACG TCA CCA ATG AAA AAG GAA TT TTT TTT TCC TCC TCC TTC	68
24-33-H6	AC AGC CAG CTG GCG AA AGG GGG ATG AAA TTG T TTT TTT TCC TCC TCC TTC	50

H7-42	CTT CCT CTC TTC TTT TTT TCT TCG CTA TTA CGT GCC ACG CTG TAA TGG GA	50
H7-185-H6	CTT CCT CTC TTC TTT TTT AGG CCG GAG TAA ATA TTG ACG GAA AAC GGC TA TTT TTT TCC TCC TCC TTC	68
H7-25-43-H6	CTT CCT CTC TTC TTT TTT AG GCG ATT TGG GAA GG GCG ATC GGG CAT CGT A TTT TTT TCC TCC TCC TTC	68
H7-178-H6	CTT CCT CTC TTC TTT TTT TTA AAG GTC CAG TAG CAC CAT TAC AAA ATC TC TTT TTT TCC TCC TCC TTC	68
H7-52-34-H6	CTT CCT CTC TTC TTT TTT GC AAC TGT AAG TTG GG TAA CGC CAT TCG TAA T TTT TTT TCC TCC TCC TTC	68
H7-70-52	CTT CCT CTC TTC TTT TTT TT CGC ATT TCT GCC AG TTT GAG GGC AGG CTG C	50
97-H6	TAC TAA TAG TCA AAT CAC CAT CAA TGG AGC AA TTT TTT TCC TCC TCC TTC	50
H7-114-H6	CTT CCT CTC TTC TTT TTT AAA GTA CGA TTC CCA ATT CTG CGA ATC AAT TC TTT TTT TCC TCC TCC TTC	68
H7-132	CTT CCT CTC TTC TTT TTT TAC ATA ACA GGT CAG GAT TAG AGA ATG CAA CT	50
168-H6	AGA CAG CAG GAA CCG AAC TGA CCA CCC AAA TC TTT TTT TCC TCC TCC TTC	50
H7-186-H6	CTT CCT CTC TTC TTT TTT CAA AAT CAG AAT TAT CAC CGT CAC GCA GCG AA TTT TTT TCC TCC TCC TTC	68
H7-62-80-H6	CTT CCT CTC TTC TTT TTT AT CAT ATG TCA TTG CC TGA GAG TCT ATG ATA T TTT TTT TCC TCC TCC TTC	68
80-98-H6	TC AAC CGT GAG CTG AA AAG GTG GCA CGA GTA G TTT TTT TCC TCC TCC TTC	50
H7-115-133	CTT CCT CTC TTC TTT TTT AA TTG CTC AAT ACC AC ATT CAA CTG AAG AAA A	50
H7-133-151-H6	CTT CCT CTC TTC TTT TTT AT CTA CGT AAC CGG AT ATT CAT TAA CTT TGA A TTT TTT TCC TCC TCC TTC	68

TFO-forming strands in solution.

Name	Sequences	Bases
H3	TCT TCC CTCTCTCT TTT TCT TTT TCC TTC	29
H4	ACT AGA AGG GAGAGAGA AAA AGA AAA AGG AAG TCG	35
H5	CGA CTT CCT TTT TCT TTT TCTCTCTC CCT TCT AGT	35
H8	AGG AGG AGG AAG GAA GGA GAG AAG CTC TCA	30
H9	TGA GAG CTT CTC TCC TTC CTT CCT CCT CCT	30

Table S1: DNA sequences.

	Cycle 1	Cycle 2	Cycle 3
Yield of duplex products (pH7.8)	95%	97%	97%
Yield of triplex products (pH5.0)	78%	81%	80%

Table S2: the yields of targeted products of each cycle in solution.

	Cycle 1 (pH7.8)	Cycle 1 (pH5.0)	Cycle 2 (pH7.8)	Cycle 2 (pH5.0)
Number of overall origamis	540	578	213	575
Number of misfolded origamis	23	232	106	350
Number of properly folded origamis	517	346	107	225
Number of origamis with right pattern	475	311	96	202
Effective yield of origamis with expected pattern	92%	90%	90%	90%

Table S3: the effective yields of DNA origamis with expected pattern in each cycle

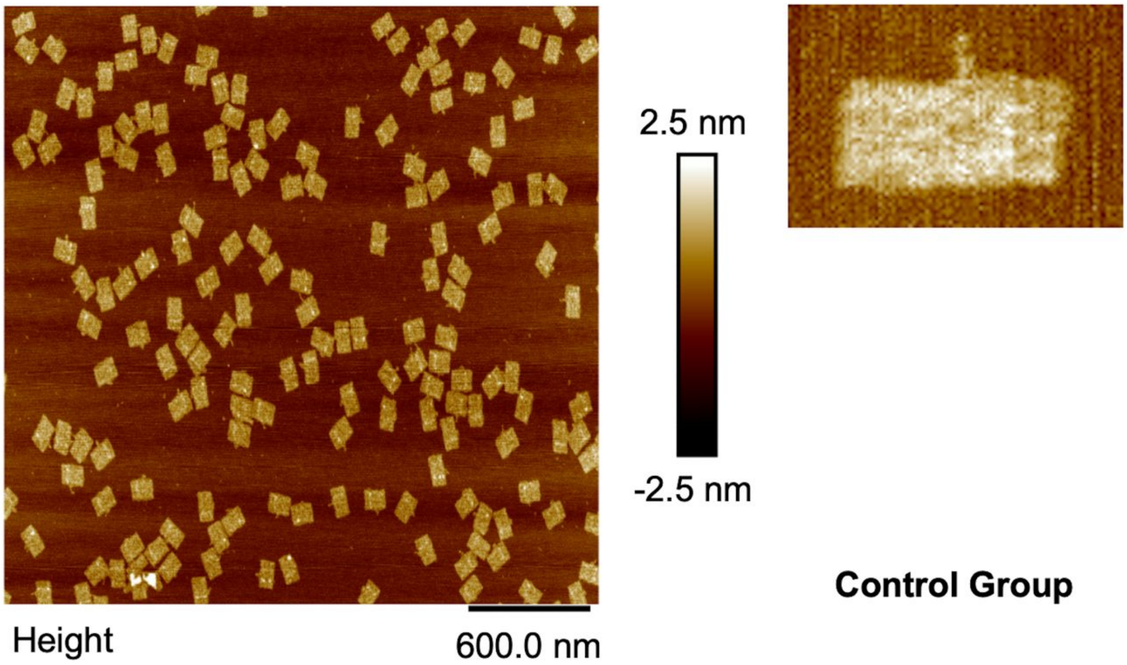

Figure S1: AFM image of control DNA origami. DNA origami with only single strand H1, H2, H6 and H7 on the anchoring sites was characterized by AFM and no obvious pattern was observed on the surface of DNA origami.

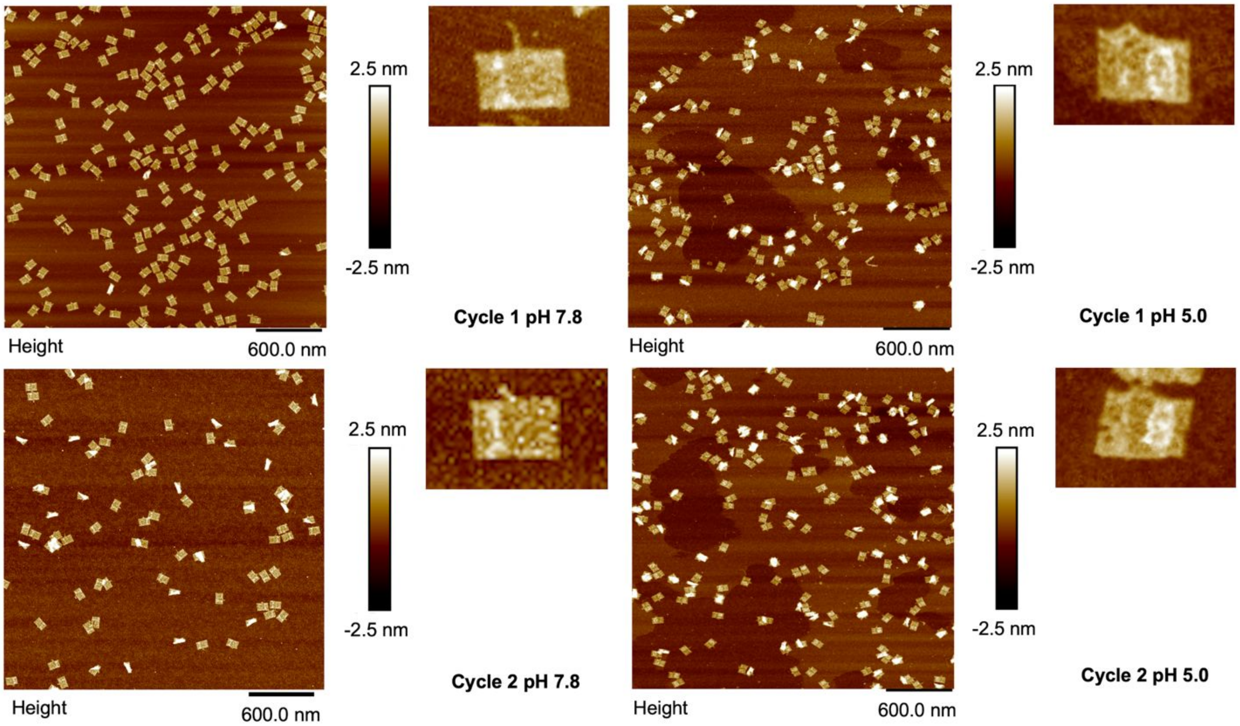

Figure S2: AFM images of cycling of duplex-triplex interconversion devices on DNA origami. DNA origami with duplex-triplex interconversion devices was annealed at pH 7.8 at first, switched to pH 5.0, switched back to pH 7.8 and switched to pH 5.0 again. AFM images of four stages were displayed respectively.
